# Distribution and Abundance of Archaeal and Bacterial Ammonia Oxidizers in the Sediments of the Dongjiang River, a Drinking Water Supply for Hong Kong

**DOI:** 10.1264/jsme2.ME13066

**Published:** 2013-11-19

**Authors:** Wei Sun, Chunyu Xia, Meiying Xu, Jun Guo, Aijie Wang, Guoping Sun

**Affiliations:** 1 School of Bioscience and Bioengineering, South China University of Technology, Guangzhou 510070, China; 2 Guangdong Provincial Key Laboratory of Microbial Culture Collection and Application, Guangdong Institute of Microbiology, Guangzhou 510070, China; 3 Harbin Institute of Technology, Harbin 150090, China; 4 State Key Laboratory of Applied Microbiology, South China (The Ministry-Province Joint Development), Guangzhou 510070, China

**Keywords:** Dongjiang River, Ammonia-oxidizing archaea (AOA), Ammonia-oxidizing bacteria (AOB), *amoA* gene, river sediment

## Abstract

Ammonia-oxidizing archaea (AOA) and bacteria (AOB) play important roles in nitrification. However, limited information about the characteristics of AOA and AOB in the river ecosystem is available. The distribution and abundance of AOA and AOB in the sediments of the Dongjiang River, a drinking water source for Hong Kong, were investigated by clone library analysis and quantitative real-time PCR. Phylogenetic analysis showed that Group 1.1b-and Group 1.1b-associated sequences of AOA predominated in sediments with comparatively high carbon and nitrogen contents (*e.g.* total carbon (TC) >13 g kg^−1^ sediment, NH_4_^+^-N >144 mg kg^−1^ sediment), while Group 1.1a- and Group 1.1a-associated sequences were dominant in sediments with opposite conditions (*e.g.* TC <4 g kg^−1^ sediment, NH_4_^+^-N <93 mg kg^−1^ sediment). Although *Nitrosomonas*- and *Nitrosospira*-related sequences of AOB were detected in the sediments, nearly 70% of the sequences fell into the *Nitrosomonas*-like B cluster, suggesting similar sediment AOB communities along the river. Higher abundance of AOB than AOA was observed in almost all of the sediments in the Dongjiang River, while significant correlations were only detected between the distribution of AOA and the sediment pH and TC, which suggested that AOA responded more sensitively than AOB to variations of environmental factors. These results extend our knowledge about the environmental responses of ammonia oxidizers in the river ecosystem.

Nitrification, the sequential oxidation of ammonia to nitrite and then nitrate by phylogenetically and physiologically distinct microorganisms, is a critical step in the nitrogen cycle. Ammonia-oxidizing bacteria (AOB) have been suggested as indicators of specific environmental conditions due to their virtues of wide distribution and important environmental significance (39). However, in the last few years, the findings of a key functional gene (ammonium monooxygenase, *amoA*) in *Crenarchaeota* (52) and the cultivation of a novel ammonia-oxidizing marine archaea *Nitrosopumilus maritimus* provided substantial evidence that ammonia-oxidizing archaea (AOA) play significant roles in nitrification (27). Many reports have found that AOA are numerically dominant over AOB in various environments (1, 5), and predicted that AOA played important roles in the energy flow and element cycling of the natural environment (15, 21). However, more and more evidence has shown that the abundance of AOA was lower than AOB in sediment environments (24, 37, 64). So far, reliable information on whether AOA or AOB is significantly responsible for ammonia oxidation is still controversial (17, 23, 40) and the contributions likely depend on different environments (55).

The investigation of environmental factors shaping the ecological niches of ammonia oxidizers could further broaden our understanding about the roles of AOA and AOB in nitrogen and carbon cycles. A number of environmental factors, including pH (19, 46), temperature (4, 49), salinity (13, 43), and organic matter (18, 22) as well as sulphide and phosphate (12), have been identified as important factors affecting the diversity, abundance and distribution of AOA and AOB in various ecosystems. Although the river ecosystem plays important roles in biogeochemical storage and the transformation of nitrogen material, only limited information about the distribution of ammonia-oxidizing microorganisms, as well as their relationships with environmental factors, have been reported (32, 33). Long-term geochemical signals of different environmental conditions, such as the composition of organic matter and nitrogen nutrients, and large fluctuations of various hydrological and chemical conditions occurred in river intertidal sediments (62). Therefore, it is essential to understand the distributions of AOA and AOB in these sediments and how they respond to changes in chemical properties along the river, so as to provide insights into the roles of ammonia-oxidizing microorganisms in the nitrification of the river ecosystem.

The Dongjiang River, originating in Xunwu County of Jiangxi province, is a tributary of the Pearl River and a drinking water supply for Hong Kong and several important cities in southern China. In this monsoon-dominant climate region, the spatial and temporal distributions of environmental conditions in this basin present considerable variations (57). In recent years, ammonium-nitrogen contamination has been identified as the main source of water quality deterioration in Dongjiang river with the growing population size, rapid development of urbanization, and enhanced agriculture production (57). However, the overall water quality of the river is generally above the drinking water quality standard (Chinese National Water Quality Grade II) (29, 33), which indicates that highly efficient nitrogen removal may occur in the river.

In our previous study, we investigated the abundance and distributions of AOA and AOB in the water column of the Dongjiang River and found that AOA preferred aerobic and relative low-ammonia concentrations and was more abundant than AOB (33). We hypothesize that AOA will be more sensitive to environmental conditions and lower abundance than AOB in river sediments that contain lower concentrations of dissolved oxygen (DO), and higher carbon and nitrogen nutrients than water columns. To test this hypothesis, clone libraries, qPCR assays and multivariate statistical analyses were employed to analyze the distributions, diversities and abundance of AOA and AOB, as well as their influencing factors, in the sediments of the Dongjiang River in this study.

## Materials and Methods

### Site description

The sampling sites were chosen to cover the main stream of the Dongjiang River according to the extent of economic development from upstream to downstream (Fig. S1). Heiyuan (HYN) is located in the upper reaches of the Dongjiang River where the tourism industry has been developed. Guzhu (GZN) is located in the midstream of the Dongjiang River and is a major agricultural development area. Huizhou (HZN) is located in the city with a reservoir capacity of more than 1.6 billion cubic meters, which is the main water supply for Hong Kong, Shenzhen, Guangzhou and other places, and the development of this city mainly depend on tourism, electronics and petrochemical industries. Qiaotou (QTN) is located downstream of the Dongjiang River with a water supply channeling to Hong Kong, and is mainly involved in industrial and commercial development. The tributary Xinfeng (XFN) is near the Xingfengjiang Reservoir and located in the upper branch of the Dongjiang River.

### Sample collection and environmental factor analyses

Sediments were collected in triplicate at each sampling site during the spring season (March 15th, 18th, 21st) in 2011 and stored in air-sealed plastic bottles for transportation to the laboratory. Surface sediment samples from a depth of 0–5 cm were collected from the river cross section (water depth ≈1–3 m) in an area of 5 m×5 m at the selected sampling sites. Subsamples of sediments were kept at 4°C to examine physicochemical characteristics within 48 h and −80°C for nucleic acid extraction. The pH and contents of carbon and nitrogen in the sediments were measured according to previous reports (7, 19, 34, 60).

### Potential nitrification rate

Potential nitrification rates (PNR) were measured using the chlorate inhibition method (19). Briefly, 5.0 g fresh sediment was added to 50 mL centrifuge tubes containing 25 mL phosphate buffer solution (PBS) (g L^−1^: NaCl, 8.0; KCl, 0.2; Na_2_HPO_4_, 0.2; NaH_2_PO_4_, 0.2; pH 7.4) with 1 mM (NH_4_)_2_SO_4_. Potassium chlorate with a final concentration of 10 mM was added to the tubes to inhibit nitrite oxidation. The suspension was incubated in a dark shaker at 25°C for 24 h, and then nitrite was extracted with 5 mL of 2 M KCl and determined spectrophotometrically at 540 nm with N-(1-naphthyl) ethylenediamine dihydrochloride. Apparent potential nitrification rates were calculated from the linear increase in concentrations of nitrite-N against time.

### DNA extraction and clone library construction

DNA was extracted according to the protocol reported previously (33). Archaeal *amoA* gene fragments (635 bp) were amplified by primers Arch-*amoA*F and Arch-*amoA*R (14), and bacterial *amoA* gene fragments (491 bp) were amplified by *amoA*-1F and *amoA*-2R (42). The amplification reaction and process were performed as described by Liu *et al.* (33).

After PCR amplification of the archaeal and bacterial *amoA* genes, gel slices of an agarose gel containing the PCR products (triplicate PCR products for each sample to minimize PCR bias) were excised and purified using the Agarose Gel DNA Purification Kit Ver. 2.0 (Takar, Dalian, China). The purified PCR products were linked into the pMD19 T-vector (Takara) and transformed into competent *E. coli* DH_5α_ cells. White colonies were selected, transferred to LB-ampicillin plates and incubated overnight. PCR amplifications with the primer set M13-47 and RV-M were used to confirm the insertion of an appropriate-sized DNA fragment. PCR products were checked by 1% (w/v) agarose gel electrophoresis. Clones with the expected insert were sequenced using M13-47 on an ABI 3730 automated sequencer.

### Sequence and phylogenetic analysis

DNAStar version 7.1.0 was used to assemble and edit nucleotide sequences. To describe similarities and differences between the community structures, the shared and unique operational taxonomic units (OTUs) defined by 3% differences in nucleotide sequences were calculated by Mothur v. 1.24.0 (45). The biodiversity (Shannon, Simpson) and richness indicators (Chaol and S_ACE_) were also obtained using Mothur software. Representative sequences of archaeal and bacterial *amoA* genes from each OTU were selected and aligned using the MEGA 5.05 software package. Phylogenetic trees were constructed with the neighbor-joining method and bootstrap analysis was used to estimate the confidence of the tree topologies using the Kimura-2-parameter distance for 1,000 replicates (47).

### Quantitative PCR assay of the sediment *amoA* genes

All qPCR assays targeting *amoA* genes in sediment samples were determined in triplicate using a Mastercycler ep real plex4 (Eppendorf, Hamburg, Germany). Copies of archaeal *amoA* and bacterial *amoA* genes were determined using primers reported previously (14, 42). Each reaction was performed in a 25 μL volume containing 1 μL DNA template, 1 μL bovine serum albumin (BSA) (100 mg mL^−1^; Roche, Indianapolis, IN, USA), 1 μL of each primer (10 μM) and 12.5 μL Power SYBR-Green PCR Master Mix (Takara). The PCR protocols were 30 s at 53°C (for AOA) or 30 s at 55°C (for AOB), and 1 min at 72°C, with readings taken between each cycle. The negative control containing no DNA templete was used during the same procedure to exclude any possible contamination. The specificity of the qPCR assay was confirmed by melting curve analysis (60–95°C, 0.1°C per read, 8 s hold) and checking with agarose gel electrophoresis.

We used the constructed plasmids containing the targeted gene sequences (archaeal and bacterial *amoA*) as standards, and plasmids were extracted with the TIANprep Mini Plasmid Kit (Tiangen, Beijing, China). The copy number of standard plasmids with tenfold serial dilutions could be calculated by their concentrations. The abundance of targeted genes in each sample was calculated by parallel quantitative PCR of the standard plasmids. The qPCR amplification efficiencies were 102% (AOA *amoA*) and 107% (AOB *amoA*), and correlation coefficients (*R*^2^) were greater than 0.99 for the targeted genes. All PCR reactions were analyzed in triplicate.

### Statistical analysis

Hierarchical clustering of environmental factors was conducted with Minitab statistical software (release 16; Minitab, State College, PA, USA) using normalized environmental data (i.e., adjusted to 0 mean and 1 standard deviation [SD] via Z transformation) (11). One-way analysis of variance (ANOVA) followed by the Student-Newman-Keulstest test was used to check for sediment physicochemical properties and community abundance using SPSS version 17.0 software. The community structures of archaeal and bacterial *amoA* genes between any two clone libraries were compared with LIBSHUFF software (44). To further rigorously examine community classification of the AOA and AOB assemblages, hierarchical clustering analysis with the Jclass supporting values was conducted using Mothur v. 1.24.0. The correlations between the environmental variables and community abundance were identified with Pearson’s moment correlation using SPSS version 17.0. Redundance analysis (RDA) was carried out to analyze the correlations between AOA and AOB community distributions with environmental parameters using Canoco software version 4.5 (48).

### Nucleotide sequence accession numbers

Sequences obtained in this study have been deposited in GenBank under accession numbers JN786985–JN787054 for AOA and JN787055–JN787099 for AOB.

## Results

### Physicochemical characteristics of sediments

The major properties of the sediments along the Dongjiang River have been shown in our previous report (53). In this river, sediment pH ranged from 5.8 to 6.7. The concentrations of sediment ammonium nitrogen varied from 39.26 to 287.63 mg kg^−1^ and were obviously higher than nitrite and nitrate. Sediments of XFN and GZN exhibited significantly lower concentrations of nitrogen (*p*<0.05, especially ammonium nitrogen) than HYN, HZN, and QTN. Variations of total carbon (TC) were from the lowest concentration 3.0 g kg^−1^ of XFN to the highest concentration 21.0 g kg^−1^ of HZN. The water contents (WC) of XFN and GZN were similar (28–32%) and significantly different from HYN, HZN, and QTN (48–52%) (*p*<0.05). Overall, the contents of sediment nitrogen and carbon were lower in XFN and GZN than in HYN, HZN and QTN. Moreover, multivariate clustering of the physicochemical factors in Dongjiang River identified two clusters of environmental conditions: sites XFN and GZN were grouped together, and the remaining sites HYN, HZN and QTN were grouped together (Fig. S2).

PNR ranged from 0.047 to 0.199 μg NO_2_^−^-N (g dry sediment)^−1^ h^−1^. HZN showed the highest PNR, followed by QTN, HYN, GZN, and XFN. Moreover, the PNR in HZN was 1.5–7.1 times greater than the other sediment samples. Positively significant correlations were found between sediment pH, TC and PNR (*p*<0.05), suggesting that pH and TC had impacts on the PNR.

### Diversity and phylogenetic analysis of sediment AOA and AOB communities

**Fig. 1:**
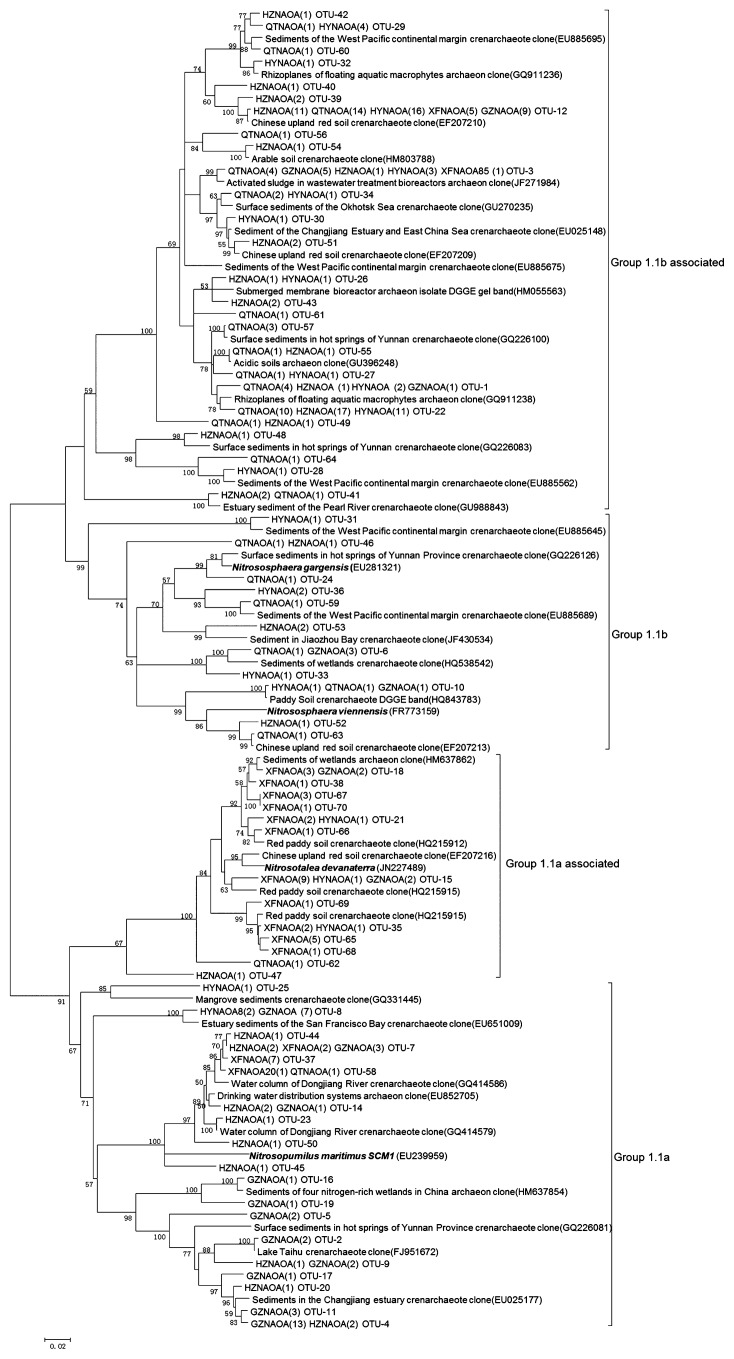
Phylogenetic tree of the archaeal *amoA* gene sequences (JN786985–JN787054) from sediments of the Dongjiang River. Note: This den-drogram was constructed by the NJ method; all reference sequences were obtained from GenBank; two capital letters and six numbers in brackets represent the sequence accession number; the clones were designated by sample name and the numbers in brackets represent the number of clones in each sample; the numbers close to the nodes represent bootstrap values of ≥50% (*n=*1,000 replicates); scale bar represents 0.02 nucleic acid substitutions per nucleotide position. XFN = Xinfeng; HYN = Heyuan; GZN = Guzhu; HZN = Huizhou; QTN = Qiaotou.

A total of 279 archaeal *amoA* sequences were retrieved from AOA clone libraries and 17 to 28 OTUs of AOA were recovered from the five sediment samples based on a 3% sequence dissimilarity cutoff (Table S1). Shannon indexes were HZN >GZN >QTN >XFN >HYN (Table S1). Therefore, AOA showed higher diversities in the downstream sampling sites (GZN, HZN and QTN) than in the upstream sampling sites (XFN and HYN). The phylogenetic tree was constructed with archaea OTU representative sequences in this study and the relative sequences deposited in Genbank (Fig. 1). Most of the archaeal *amoA* gene sequences from HYN (88.7%), HZN (79.0%), and QTN (96.2%) were attached to Group 1.1b- and Group 1.1b-associated sequences (Fig. S3a), which were affiliated with the sequences retrieved from sediments of the West Pacific, Changjiang, the Pearl River Estuary and hot springs (9, 10, 25, 26), and Chinese upload red soil or arable soils (16, 19). In contrast, Group 1.1a-associated sequences were dominated exclusively by XFN (68.0%), which were related to *amoA* sequences from paddy fields, red paddy or Chinese upland red soil (7, 19, 54). Sequences in Group 1.1a were dominated exclusively by GZN (60.0%), which shared high similarity with sequences from water columns or drinking water distribution systems (33, 51), and sediments of mangroves, estuaries, lakes, or hot springs (10, 25, 31, 37, 58). These results suggested that the community compositions of AOA varied among the sediment samples.

**Fig. 2:**
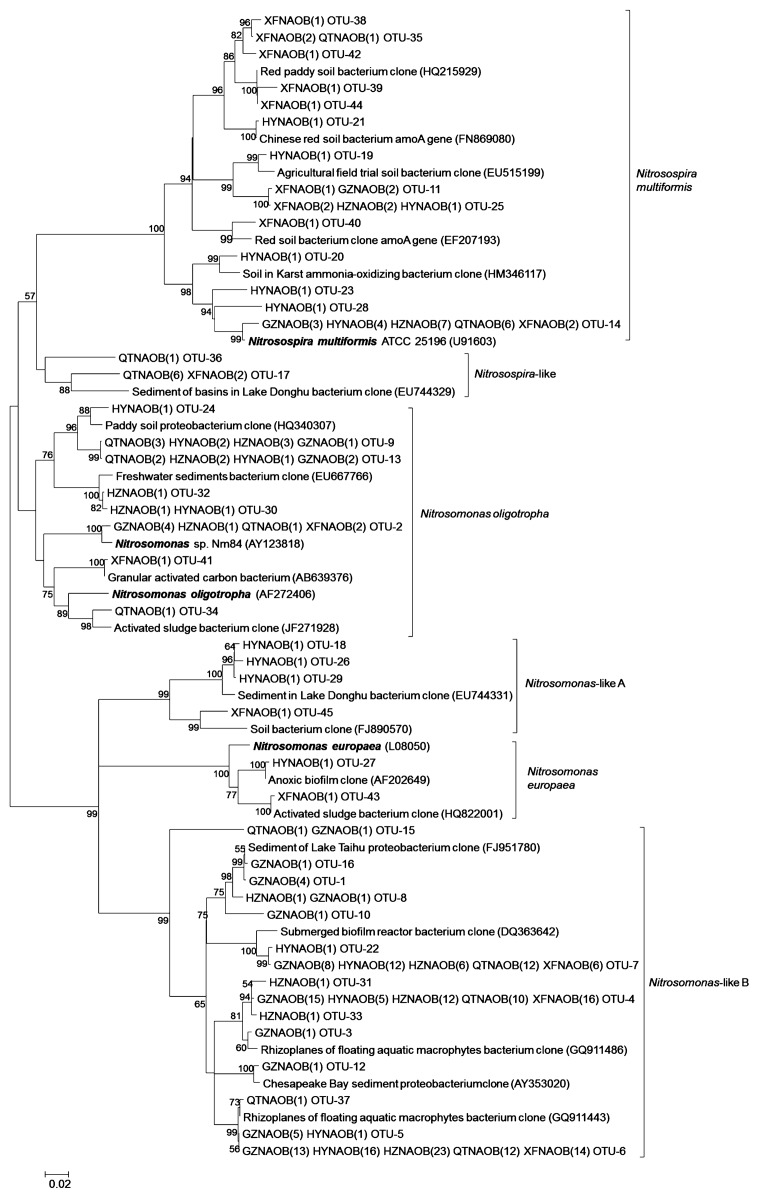
Phylogenetic tree of the bacterial *amoA* gene sequences (JN787055–JN787099) from sediments of the Dongjiang River. Note: This den-drogram was constructed by the NJ method; all reference sequences were obtained from GenBank; two capital letters and six numbers in brackets represent the sequence accession number; the clones were designated by sample name and the numbers in brackets represent the number of clones in each sample; the numbers close to the nodes represent the bootstrap values of ≥50% (*n=*1,000 replicates); scale bar represents 0.02 nucleic acid substitutions per nucleotide position. XFN *=* Xinfeng; HYN *=* Heiyuan; GZN *=* Guzhu; HZN *=* Huizhou; QTN *=* Qiaotou.

Overall, 290 clones for bacterial *amoA* were randomly selected for sequencing from all sediment samples, and 13 to 20 OTUs were recovered from each clone library based on 3% sequence dissimilarity cutoff (Table S1). The highest OTU number of AOB occurred at HYN, followed by XFN, GZN, QTN, and HZN, consistent with the values of Shannon and Chaol indices (Table S1). Overall, AOB had higher diversities in the upstream sites of XFN, HYN and GZN than the downstream sites of HZN and QTN. The recovered sequences from the sediments were associated with known genera, *Nitrosomonas* and *Nitrosospira* (Fig. 2). About 14.5% bacterial *amoA* gene sequences from all sediment samples fell into the *Nitrosospira multiformis* cluster (Fig. S3b), which were affiliated with sequences from soil environments, such as red soil, agriculture field soil and vegetation restoration soil in Karst (7, 36, 38, 61). The sequences distributed in the *Nitrosospira*-like cluster were only recovered from XFN and QTN, which were related to the sequences from sediments of Lake Donghu (6). Most sequences received from all sediment samples (XFN 74.6%, HYN 83.4%, GZN 92.1%, HZN 85.2%, QTN 75.5%) were affiliated to *Nitrosomonas* genera (Fig. S3b), and they were further divided into four different clusters (Fig. 2). Members of the *Nitrosomonas oligotropha* cluster were distributed in all sediment samples and were closely affiliated to the sequences from activated sludge, granular active carbon, freshwater sediment and paddy soil (20, 63). The clusters of *Nitrosomonas*-like A and *Nitrosomonas europaea* only contained a small proportion of sequences (2.4%) recovered from XFN and HYN. A large proportion of sequences (69.3%) recovered from all sediment samples belonged to *Nitrosomonas*-like B cluster, and shared 87–99% similarity with the sequences recovered from Lake Taihu, Chesapeake Bay, submerged biofilm and rhizoplanes of floating aquatic macrophytes (13, 41, 56, 58).

### Community distributions of AOA and AOB in sediments along the Dongjiang River

According to the *p* values calculated by LIBSHUFF software, the community structure of AOA showed similar compositions in HYN, HZN and QTN, which were significantly different from the communities recovered from sites XFN and GZN based on pairwise comparison (Table 1). Hierarchical clustering analysis separated the AOA communities into two slightly different groups (Fig. S4a). XFN and GZN were grouped together and separated from HZN, HYN and QTN, consistent with the results of LIBSHUFF analysis. Moreover, this classification of the AOA assemblages correlated with the classification of the sediment environments in the Dongjiang River. However, for the AOB community structure, there was a significant compositional overlap among the five bacterial *amoA* clone libraries (95% confidence). Almost all of the sampling sites showed similar bacterial *amoA* compositions with the exception of samples from XFN vs GZN (Table 1), which supported minor changes of AOB composition structure along the river. Hierarchical clustering analysis showed that AOB communities were divided into two groups with XFN and QTN grouped together and the other samples clustered together (Fig. S4b).

### Abundance of *amoA* genes of AOA and AOB

**Fig. 3:**
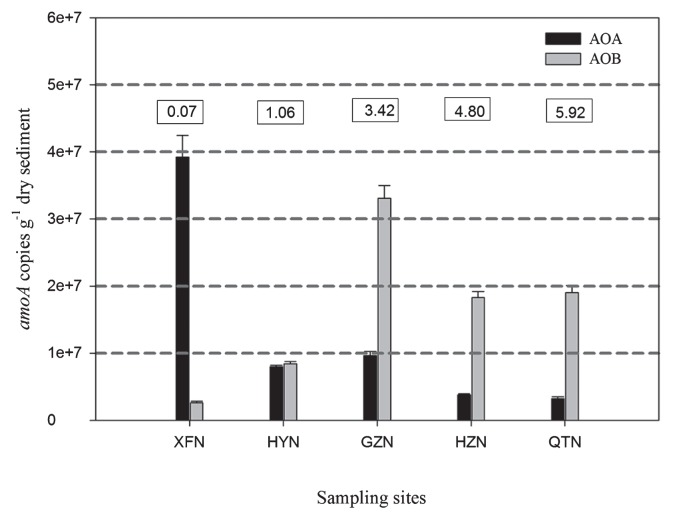
Abundance of AOA and AOB *amoA* gene copies in sediments at the Dongjiang River sampling sites, XFN, HYN, GZN, HZN and QTN. Error bars represent standard errors of triplicate samples. The ratios of bacterial to archaeal amoA copy number are shown in boxes above the chart. XFN = Xinfeng; HYN = Heiyuan; GZN = Guzhu; HZN = Huizhou; QTN = Qiaotou.

The abundance of AOA and AOB was determined by targeting the *amoA* gene. AOA *amoA* gene copy numbers ranged from 3.21×10^6^ to 3.92×10^7^ copies per gram of sediment dry weight, and the highest AOA abundance was detected in XFN, followed by GZN, HYN, and HZN. The lowest AOA abundance appeared in QTN, which was 12.2 times lower than XFN (Fig. 3). Pearson correlation analysis showed that the pH of the sediments was significantly negatively correlated with the abundance of AOA (*p*<0.05). In contrast, the abundance of the AOB *amoA* gene showed a different trend. The copy numbers of the AOB *amoA* gene varied from 2.62×10^6^ to 3.31×10^7^ copies per gram of sediment dry weight and were significantly higher in GZN than any other samples, and lowest in XFN (Fig. 3). There was no significant difference of AOB abundance between HZN and QTN. The relative *amoA* gene abundance of AOB to AOA ranged from 0.07±0.01 to 5.92±0.11 (Fig. 3). The *amoA* copy numbers of AOB were greater than those of AOA along the river, except for the tributary sample from XFN. The ratios of AOB to AOA increased significantly (*p*<0.05) from upstream HYN to downstream QTN, and were significantly positively correlated with the concentration of nitrite (*p*<0.01). However, no significant relationships were observed between PNR and the abundance of AOA and AOB.

### Relationships between ammonia-oxidizer communities and environmental factors

**Fig. 4:**
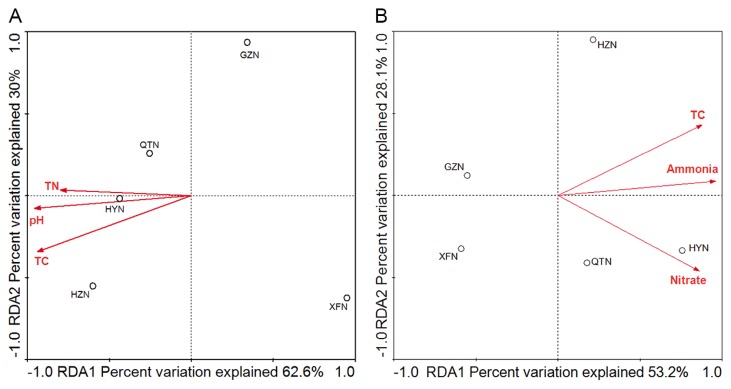
Bioplot of (Redundance analysis, RDA) correspondence analysis showing the relationship between the distribution of archaea (A) and bacteria (B) *amoA* gene OTUs derived from the clone library from XFN, HYN, GZN, HZN and QTN in the Dongjiang River and the sediment properties analyzing using CANOCO software. Correlations between RDA axes and environmental variables are represented by the length and angle of arrows (environmental vectors). ○ sampling sites; → Environmental factors. XFN = Xinfeng; HYN = Heiyuan; GZN = Guzhu; HZN = Huizhou; QTN = Qiaotou.

RDA was used to elucidate the correlations between environmental factors and community structures of AOA and AOB. For the AOA community, pH, TC and TN were selected based on the forward selection process and variance inflation factors with 999 Monte Carlo permutations. These environmental factors could explain 79.1% of total variance by the first two RDA axes (Fig. 4A). The cumulative variance of the AOA community-environment relationship was explained 62.6% by the first axis and 30% by the second axis (Fig. 4A). Moreover, pH (*p=*0.021) and TC (*p=*0.001) were significantly correlated with the distribution of the AOA community, which contributed 65.3% to the total RDA explanatory power. Although the concentrations of ammonium, nitrate, and TC were identified as the top three highest loading environmental variables for the distribution of AOB with 53.2% and 28.1% explanations by the first and the second axes, respectively (Fig. 4B), no significant correlation was observed between AOB distribution and the environmental parameters.

## Discussion

Although higher abundance of AOA relative to AOB has been observed in many habitats, such as soil (19), ocean (3) and artificial environments (28), distinctly different results of the relative abundance of AOA and AOB have been reported in freshwater sediments, and the influencing environmental factors are not well known. It was found that AOA were far more abundant than AOB in the sediments of the Qiantang River, Lake Taihu, freshwater lakes in northwest Germany and the Pearl River Estuary (20, 26, 32, 58), while higher AOB abundances were observed in the sediments of the Chongming eastern tidal flat, Qinghai Lake, and mangrove (24, 31, 64). Different environmental factors, such as pH, organic substances, and ammonia concentration, were identified as influencing factors in the relative abundance of AOA and AOB in different environmental sediments (32, 58, 64). In this study, higher abundance of AOB than AOA was detected and the relative abundance of AOB to AOA significantly positively correlated with the concentration of nitrite, which suggested that AOB was more competitive than AOA under high nitrite conditions. Incubation experiment with samples from the North Sea have already shown the possibly toxic effect of a high nitrite concentration on marine *Crenarchaeota*, although the abundance of marine *Crenarchaeota* correlated positively with nitrite (59).

In this study, AOB outnumbered AOA in almost all of the sediment samples in Dongjiang River (Fig. 3), which was different from our previous observation of higher AOA abundance than AOB in water samples collected in summer 2008 (33). Similar results were reported in Qinghai Lake, where AOA were more abundant than AOB in oxic lake water; the opposite was true in anoxic sediments (24). The different survival mechanisms of AOA and AOB under different redox conditions in the anoxic sediments and the oxic water columns may partially explain the differential distributions of AOA and AOB abundance (24). The inverted environmental profile was the same as the lake: oxic water and anoxic sediments, and concentrations of nitrogen and carbon contents in the sediments were significantly higher than those in the water columns. Based on the lower half-saturation constant (*Km*) for *Thaumarchaeota* (35), AOA could prefer aerobic and relative low-ammonia concentration conditions and better adapt to oligotrophic environments (33). Additionally, large seasonal variation of rainfall and runoff within the Dongjiang River basin, which has a subtropical climate and is dominant in front- and typhoon-type rainfall, may also cause the dynamic changes of AOA and AOB among different sampling seasons.

Although positive relationships between pH and the abundance of AOA were discovered in acid soils (pH 3.7–5.8) (19) and the sediments of Lake Taihu (pH 6.8–8.0) (58), significantly negative correlations (*p*<0.05) were detected in this study in which the sediment pH ranged from 5.8 to 6.7, which was consistent with the observations in the sediments along the Qiantang River (pH 6.5–8.0) (32). The reasons for the inconsistency of the effect of pH on AOA may be attributed to the different sediment or soil types and the pH variation ranges. Strikingly, the classified results of the AOA community structure correlated with the sediment parameters along the river. Group 1.1b- (*Nitrososphaera*) and Group 1.1b-associated (*Nitrososphaera*-associated cluster) were the dominant sequences in HYN, HZN and QTN where the contents of TC (13.2–21.0 g kg^−1^ sediment) and ammonium (144.2–287.6 mg kg^−1^ sediment) were significantly higher than those in XFN and GZN (TC: 3.0–3.4 g kg^−1^ sediment; ammonium: 39.3–92.2 mg kg^−1^ sediment) (Fig. 1 and Fig. S3a). These results were expected since *Nitrososphaera*- and *Nitrososphaera*-associated clusters could bear higher amounts of organic carbon (8, 32, 50) and ammonium nitrogen than *Nitrospumilus and Nitrosotalea* (32). Similar results were also found in the sediments of the Qiantang River, where organic carbon contents of sediments (13.4–34.2 g kg^−1^ sediment) were higher than those of the Dongjiang River and more than 90% sequences belonged to *Nitrososphaera*- and *Nitrososphaera*-associated clusters (32). Additionally, GZN was dominated by Group 1.1a (*Nitrospumilus*), which could be inhibited by organic carbon (27) and prefer relatively lower amounts of carbon content than *Nitrososphaera*, while Group 1.1a-associated (*Nitrosotalea*), an obligate acidophilic ammonia oxidizer and consistent with the *Nitrospumilus* in its affinity of ammonium (30), was dominant in XFN, where the pH (5.76) and ammonium content were the lowest among the sediment samples (Fig. S3a). RDA analyses in the present study further confirmed that pH and TC were key regulators of the distribution of AOA (Fig. 4A). Similar results were discovered in the Qiantang River, where pH and organic carbon and ammonium contents were identified as important factors that affected the contribution of AOA to nitrification (32). These environmental factors seem to be important in influencing the distribution of the AOA community in the river ecosystem. In contrast, minor changes of AOB were observed along the river and nearly 70% sequences clustered into an unknown *Nitrosomonas*-like B cluster, which may represent the dominant source of the autochthonous and even novel AOB lineage in the Dongjiang River (Fig. 2 and Fig. S3b). However, no significant correlations were observed between the abundance and distribution of AOB and the environmental factors in this study, which was consistent with the results in Lake Taihu (58). These results suggested that AOA responded more sensitively than AOB to different environmental factors.

The correlation between the PNR and *amoA* gene abundance of AOA and AOB may be helpful to compare their relative contributions to microbial ammonia oxidation under different ecosystems and heterogeneous environmental conditions. Zheng *et al.* (64) found that PNR were significantly greater in summer than in winter and correlated strongly with the abundance of *amoA* genes in Chongming eastern intertidal sediments. In contrast, AOA abundance did not correlate with PNR, although it was found to be always greater than that of AOB along an estuarine salinity gradient (2). PNR in the present study were significantly higher in HYN, HZN, and QTN than those in XFN and GZN. The lack of correlations between PNR and the abundance of AOA and AOB has several possible explanations. Since PNR are typically measured under unlimited oxygen and substrate conditions (2), substrate concentrations or other conditions of PNR experiments might not be very appropriate or optimized for the activity measures of AOA and AOB. In addition, the roles of heterotrophic nitrifiers that are capable of ammonia oxidation under certain environmental conditions have not yet been accounted for oxidizing ammonia. Therefore, activity-based analyses of ammonia oxidizers should be emphasized to explore and determine to interpret the complex changes of ammonia-oxidizing communities in response to dynamic environmental properties in the future.

In conclusion, the abundance of AOA was lower than that of AOB in the sediments of the Dongjiang River (from upstream to downstream), although higher AOA abundance was detected in the water column of this river. However, the community distribution of AOA was significantly correlated with sediment pH and TC, while no environmental factor was observed to influence the abundance and distribution of the AOB community. These results suggested that AOA changed more sensitively than AOB to the fluctuation of environmental factors. Further studies using the stable-isotope probing (SIP) method combined with metatranscriptomes are needed to elucidate the activities and mechanisms of AOA and AOB in river sediments.

## Supplementary Information



## Figures and Tables

**Table 1:** Comparisons of archaeal and bacterial *amoA* gene clone libraries in the sediments of the Dongjiang River

Group	Homologous *X* library sites	*p* values for heterologous library *Y* sites				

		XFN	HYN	GZN	HZN	QTN
AOA	XFN	—	<0.0001	<0.0001	<0.0001	0.0001
	HYN	*0.0086*	—	<0.0001	*0.0098*	*0.9168*
	GZN	0.0021	0.0007	—	0.0007	0.0001
	HZN	<0.0001	*0.2464*	*0.0033*	—	*0.9505*
	QTN	<0.0001	*0.4768*	<0.0001	*0.0178*	—

AOB	XFN	—	*0.9415*	0.0006	*0.1534*	*0.3448*
	HYN	*0.0594*	—	*0.0338*	*0.7324*	*0.1745*
	GZN	*0.0086*	*0.3997*	—	*0.4549*	*0.0130*
	HZN	*0.0222*	*0.1909*	*0.1156*	—	*0.0102*
	QTN	*0.3430*	*0.0821*	*0.0620*	*0.3460*	—

When comparing multiple libraries, the LIBSHUFF *p* values were compared with the critical value to confirm whether the libraries were different. The critical *p* value is a=0.0026 for the five clone libraries analyzed in the present study. For each pairwise comparison, if both *p* values calculated by LIBSHUFF are higher than a (0.0026), the result indicates that there is no significant difference in the composition of the communities for the two compared clone libraries (95% confidence). *p* values set in italics indicate the two clone libraries with pairwise comparison that were not significantly different. XFN = Xinfeng; HYN = Heiyuan; GZN = Guzhu; HZN = Huizhou; QTN = Qiaotou.
