# Supplementary material for: Distribution and Abundance of Archaeal and Bacterial Ammonia Oxidizers in the Sediments of the Dongjiang River, a Drinking Water Supply for Hong Kong
Source: Microbes Environ. 2013 Nov 19;28(4):457–65. doi: 10.1264/jsme2.ME13066 (PMC4070707; doi:10.1264/jsme2.ME13066)
Supplement: Supplementary file 1 [file 28_457_s1.pdf]

Table S1 Biodiversity and predicted richness of the AOA and AOB *amoA* genes recovered from the sediments of the Dongjiang River

amoA gene	Station	No. of clones	No. of OTUs	*Shannon*	*Simpson*	S_{ACE}	*Chao1*
AOA	XFN	50	17	2.55	0.078	24.24	22.25
	HYN	53	20	2.37	0.138	99.24	50.33
	GZN	60	19	2.64	0.084	27.16	23.00
	HZN	62	28	2.75	0.105	57.25	45.00
	QTN	54	23	2.59	0.106	234.35	91.00
AOB	XFN	55	17	2.21	0.156	33.35	23.00
	HYN	54	20	2.34	0.142	168.57	72.50
	GZN	63	16	2.30	0.122	24.90	23.00
	HZN	61	13	1.94	0.197	28.74	18.00
	QTN	57	13	2.13	0.132	31.00	20.50

XFN = Xinfeng; HYN = Heiyuan; GZN = Guzhu; HZN = Huizhou; QTN = Qiaotou.

Fig. S1 Locations of five sample sites in the Dongjiang River. Qiaotou site is near the entry of water supply channel to Hong Kong.

Fig. S2 The sediment environment hierarchical clustering dendrogram constructed using Euclidean distance and Ward linkage of sediment physicochemical factors in the Dongjiang River. XFN = Xinfeng; HYN = Heiyuan; GZN = Guzhu; HZN = Huizhou; QTN = Qiaotou.

Fig. S3 Relative abundance of AOA (a) and AOB (b) *amoA* gene clone libraries at five sites of the Dongjiang River based on Fig. 2 and Fig. 3, respectively.

Fig. S4 Hierarchical clustering analysis (UPGMA algorithm using the distance between communities) for all archaeal (a) and bacterial (b) *amoA* gene sequences from the sediments of the Dongjiang River calculated by jclass calculator using the online Mothur software. XFN = Xinfeng; HYN = Heiyuan; GZN = Guzhu; HZN = Huizhou; QTN = Qiaotou.

Fig.S1

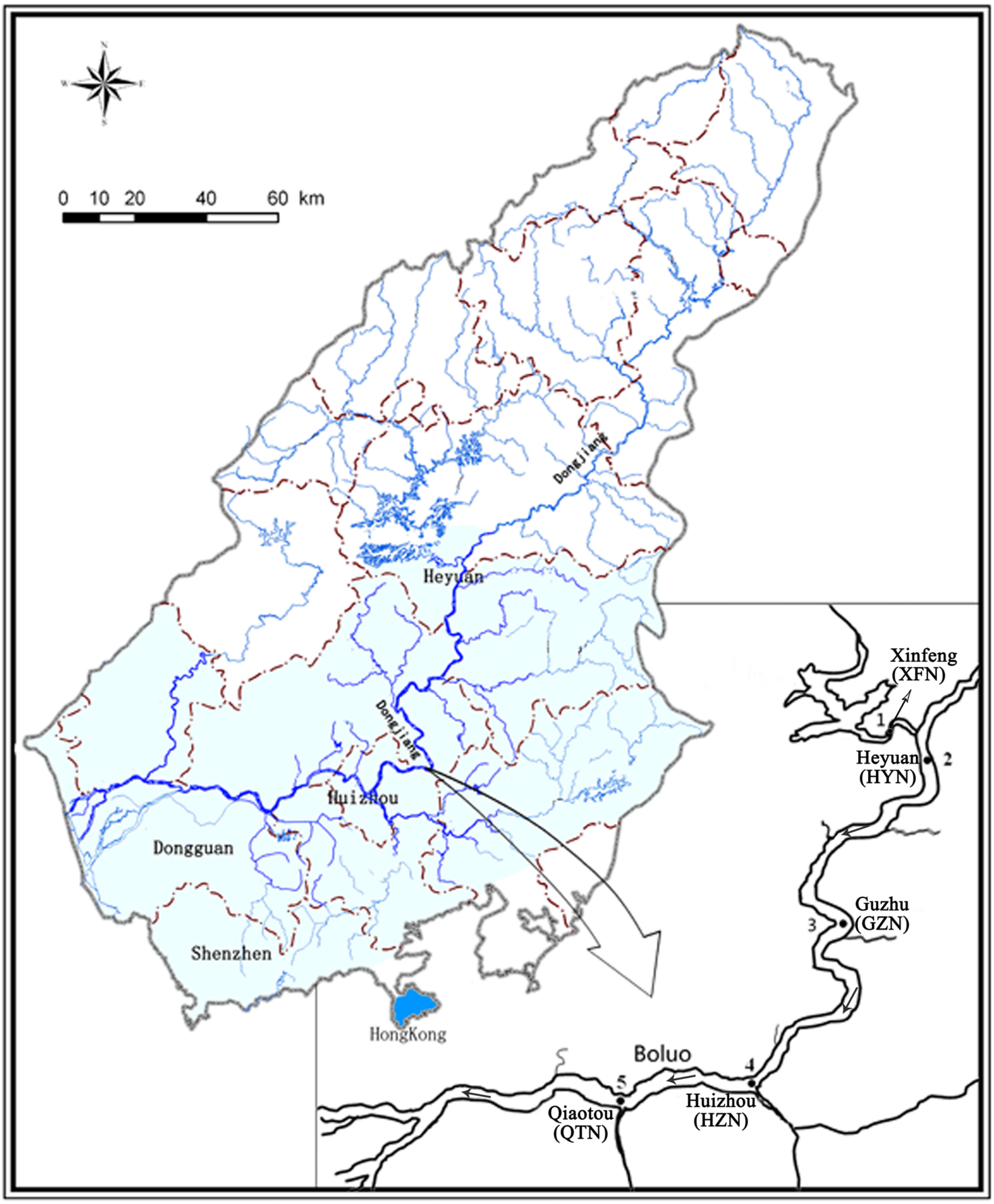

0 10 20 40 60 km
Dongjiang
Heyuan
Dongjiang
Huizhou
Dongguan
Shenzhen
HongKong
Xinfeng
(XFN)
1
Heyuan
(HYN)
2
Guzhu
(GZN)
3
Boluo
4
5
Qiaotou
(QTN)
Huizhou
(HZN)

Fig. S2

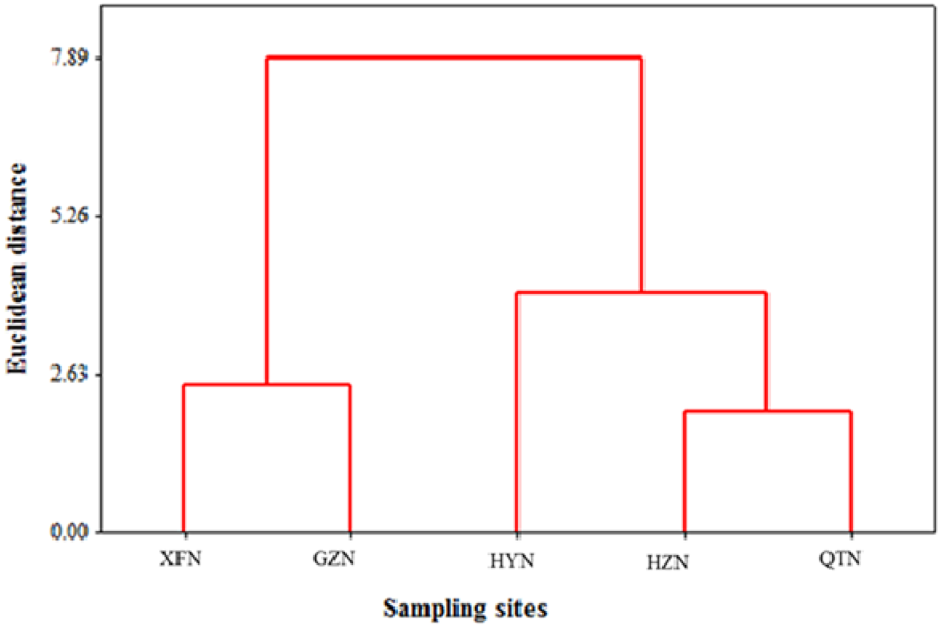
7.89
5.26
2.63
0.00
Euclidean distance
XFN
GZN
HYN
HZN
QTN
Sampling sites

Fig. S3

a

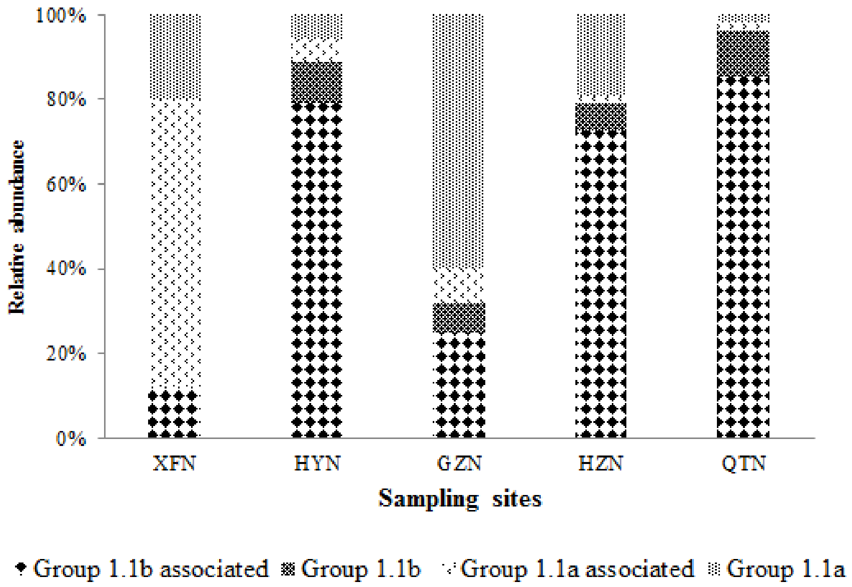
100%
80%
60%
40%
20%
0%
Relative abundance
XFN
HYN
GZN
HZN
QTN
Sampling sites
Group 1.1b associated
Group 1.1b
Group 1.1a associated
Group 1.1a

b

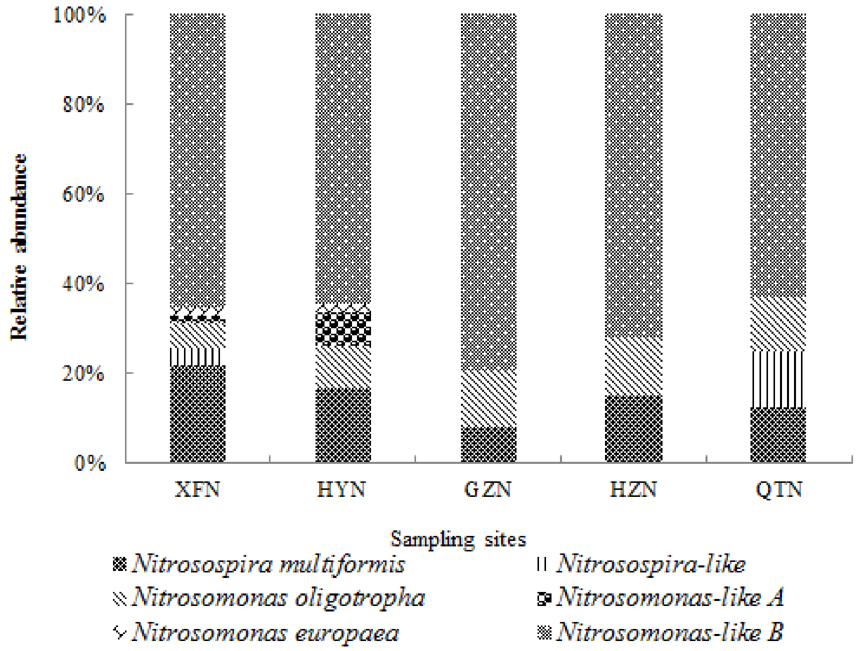
100%
80%
60%
40%
20%
0%
Relative abundance
XFN
HYN
GZN
HZN
QTN
Sampling sites
Nitrosospira multiformis
Nitrosospira-like
Nitrosomonas oligotropha
Nitrosomonas-like A
Nitrosomonas europaea
Nitrosomonas-like B

Fig. S4

a

b

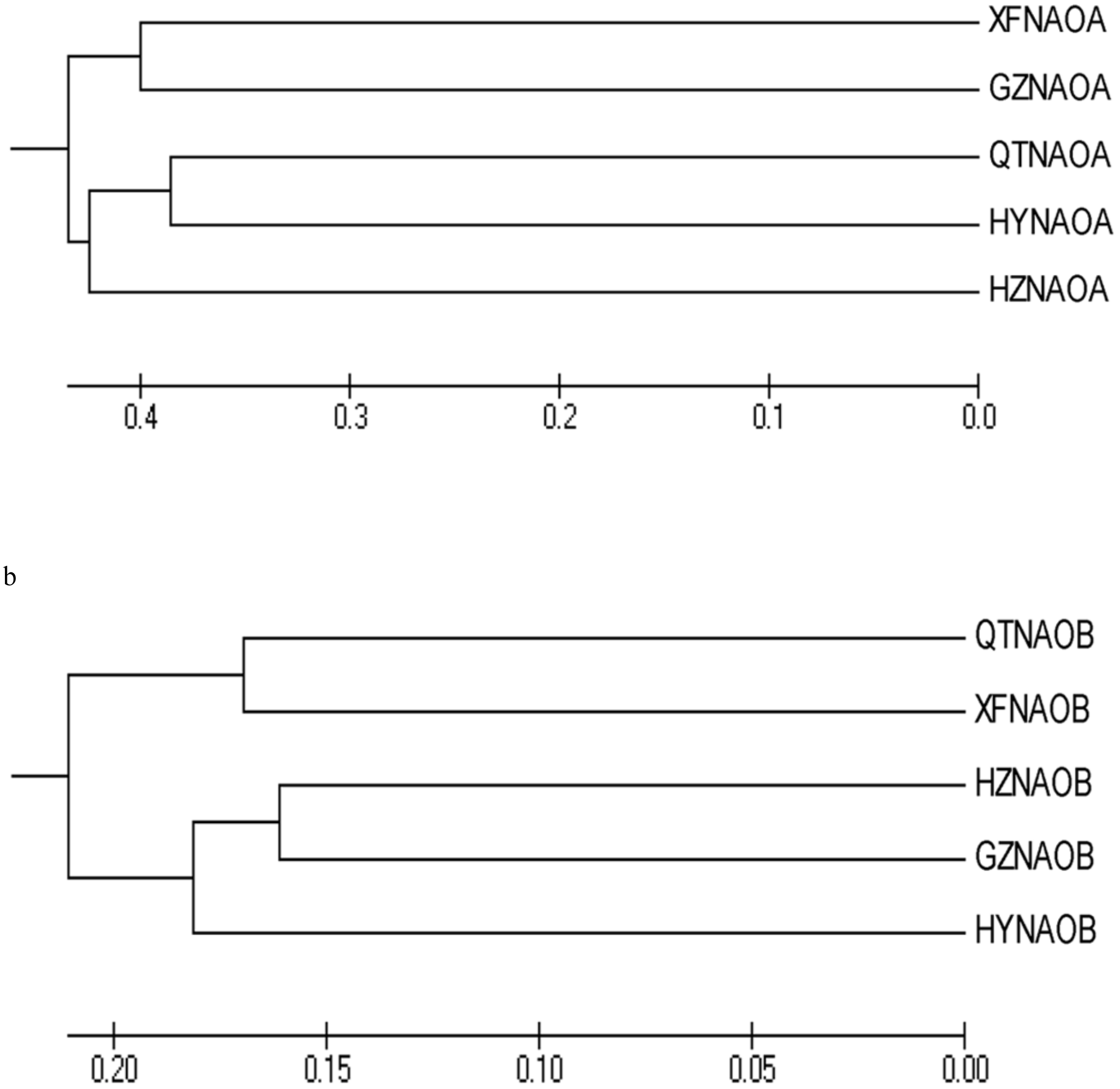

XFNAOA
GZNAOA
QTNAOA
HYNAOA
HZNAOA
0.4
0.3
0.2
0.1
0.0
QTNAOB
XFNAOB
HZNAOB
GZNAOB
HYNAOB
0.20
0.15
0.10
0.05
0.00
